# The behavioural patterns and neural correlates of concrete and abstract verb processing in aphasia: A novel verb semantic battery

**DOI:** 10.1016/j.nicl.2017.12.009

**Published:** 2017-12-06

**Authors:** Reem S.W. Alyahya, Ajay D. Halai, Paul Conroy, Matthew A. Lambon Ralph

**Affiliations:** a Neuroscience and Aphasia Research Unit, Division of Neuroscience & Experimental Psychology, Manchester Academic Health Science Centre, University of Manchester, United Kingdom; b King Fahad Medical City, Riyadh, Saudi Arabia

**Keywords:** Verbs, Concreteness, Imageability, Aphasia, Lesion-symptom mapping

## Abstract

Typically, processing is more accurate and efficient for concrete than abstract concepts in both healthy adults and individuals with aphasia. While, concreteness effects have been thoroughly documented with respect to noun processing, other words classes have received little attention despite tending to be less concrete than nouns. The aim of the current study was to explore concrete-abstract differences in verbs and identify their neural correlates in post-stroke aphasia. Given the dearth of comprehension tests for verbs, a battery of neuropsychological tests was developed in this study to assess the comprehension of concrete and abstract verbs. Specifically, a sensitive verb synonym judgment test was generated that varied both the items' imageability and frequency, and a picture-to-word matching test with numerous concrete verbs. Normative data were then collected and the tests were administered to a cohort of 48 individuals with chronic post-stroke aphasia to explore the behavioural patterns and neural correlates of verb processing. The results revealed significantly better comprehension of concrete than abstract verbs, aligning with the existing aphasiological literature on noun processing. In addition, the patients performed better during verb comprehension than verb production. Lesion-symptom correlational analyses revealed common areas that support processing of concrete and abstract verbs, including the left anterior temporal lobe, posterior supramarginal gyrus and superior lateral occipital cortex. A direct contrast between them revealed additional regions with graded differences. Specifically, the left frontal regions were associated with processing abstract verbs; whereas, the left posterior temporal and occipital regions were associated with processing concrete verbs. Moreover, overlapping and distinct neural correlates were identified in association with the comprehension and production of concrete verbs. These patient findings align with data from functional neuroimaging and neuro-stimulation, and existing models of language organisation.

## Highlights

• Developed new verb comprehension tests
• Processing of concrete verbs is more accurate than abstract verbs in aphasia
• Neural correlates of semantic verb processing distributed in left cortical regions
• Graded neural difference between processing concrete and abstract verbs
• Graded neural differences observed in production and comprehension modalities

## 1 Introduction

Words can be classified into different categories; one common classification is concrete versus abstract words (such as ‘to twist’ and ‘to exist’). Concrete concepts are more tangible, imageable and can be experienced through the senses whereas abstract words are less tangible, less imageable and they typically refer to ideas, mental or emotional states. In relation to this perspective, Paivio (1986) proposed a dual-coding theory, according to which concrete concepts benefit from dual-coding of verbal and non-verbal stores, whereas abstract concepts are represented in a verbal store only. It was assumed that the sensory and perceptual experiences associated with a concept are represented in a non-verbal store, whereas the linguistic information is stored in a verbal store. Imageability refers to the extent to which a word can conjure up a mental image and/or sensory experience. Words with high-imageability give rise to a mental image more rapidly and easily, whereas low-imageability words do so with difficulty, if at all (Paivio et al., 1968). It has been shown that concreteness and imageability are highly correlated (Paivio et al., 1968), and hence, most studies use these two terms interchangeably (though for important variations relating to high valence abstract words, see Vigliocco et al., 2014). Henceforth, the term ‘concrete’ is used to refer to high imageable concrete concepts, and ‘abstract’ for low imageable abstract concepts. The effect of concreteness on word processing has been well documented in the literature: concrete words are processed more accurate and efficiently than abstract words in healthy adults (e.g., Wiemer-Hastings and Xu, 2005), people with aphasia (e.g., Hoffman et al., 2011b, Sandberg and Kiran, 2014), and semantic dementia (e.g., Jefferies et al., 2009). This concreteness effect has also been observed in language tasks that do not place high demands on semantic knowledge, such as repetition (Tyler et al., 2000) and reading (Evans et al., 2012). These studies imply that the imageability of a word is a vital feature that supports different components of the language system including production and comprehension processes, not only at the semantic level, but also when the phonological or orthographic processes are activated. One classic explanation for these observations related to the greater semantic richness associated with concrete compared to abstract concepts (Jones, 1985, Paivio, 1986). Other theories emphasis on the greater context sensitivity for abstract items (Hoffman et al., 2011b), with convergent fMRI, TMS and neuropsychological data suggesting that both mechanisms are important and are supported by different neural networks (Goldberg et al., 2007, Hoffman et al., 2015, Hoffman et al., 2010).

Concrete-abstract differences have been explored almost entirely with respect to noun processing. Verbs vary extensively in their concreteness and imageability ratings. Concrete verbs are usually related to action and motion verbs (e.g., ‘to drink’ or ‘to walk’) whereas abstract ones are linked to cognitive and emotional verbs (e.g., ‘to process’ or ‘to care’). Moreover, the majority of words used in aphasiology, neuropsychological research and clinical practice are picture-based, constraining tests to concrete items, with a main focus on nouns. As a result, the processing of abstract and concrete verbs in aphasia remains relatively unexplored. Where verbs have been investigated it has primarily been in the context of comparing them to nouns, rather than exploring processing of different types of verbs. Bird et al. showed that imageability was a strong predictor of naming performance among individuals with verb deficits compared to those without verb deficits (Bird et al., 2001b), and among individuals with post-stroke aphasia compared to healthy controls (Bird et al., 2003).

The fact that processing concrete and abstract words can be differentially impaired suggests that there might be important, graded variations in their cognitive and neural representations. It has been argued that concrete concepts rely on sensory experiences, and thus visual and other sensory information contribute to their semantic representation (Paivio et al., 1968). On the other hand, the meaning of abstract words is more context-dependent (Schwanenflugel and Shoben, 1983) and as such might be more reliant on semantic-executive control processes (Hoffman et al., 2011b, Noppeney and Price, 2004). Accordingly, damage to the visual and sensory association regions within the ventral language pathway and particularly left temporal lobe could be expected to affect concrete but not abstract knowledge (Noppeney and Price, 2002); whereas, damage to the executive control network, including the left prefrontal cortex, could result in deficits with abstract knowledge (Hoffman et al., 2010).

A number of neuroimaging experiments have investigated differences between concrete and abstract concepts using fMRI, PET and TMS on healthy adults (e.g., Binder et al., 2005, Goldberg et al., 2007, Hoffman et al., 2015, Hoffman et al., 2010, Noppeney and Price, 2004, Perani et al., 1999, Sabsevitz et al., 2005). Studies found strong involvement of the left inferior frontal gyrus for abstract over concrete word processing. A number of other language-related regions including the left superior temporal gyrus and temporal pole have been related to abstract word processing over concrete words (Binder et al., 2005, Noppeney and Price, 2004, Perani et al., 1999, Sabsevitz et al., 2005). In contrast, the involvement of temporal (posterior inferior temporal gyrus, medial anterior temporal lobe and left inferior temporal pole) and parietal regions (posterior inferior parietal areas and angular gyrus) have been shown to be activated for processing concrete over abstract words (Binder et al., 2005, Noppeney and Price, 2002, Sabsevitz et al., 2005). Findings from these neuroimaging studies are generally consistent with the view proposing that the representation of concrete concepts are boosted by temporal and occipital areas that underpin sensory processing and visual object recognition, whereas abstract concepts rely more on frontal regions related to semantic-executive control (Breedin et al., 1998, Hoffman et al., 2015, Noppeney and Price, 2002).

To date, most functional neuroimaging experiments have utilised noun items. Few studies have investigated the neural correlates associated with low-imageability emotion and cognitive verbs in comparison to concrete motion verbs in healthy adults (Grossman et al., 2002, Rodríguez-Ferreiro et al., 2011). Where data are available, these studies have shown that processing both concrete and abstract verbs recruit left and right inferior frontal gyri. Even though these studies employed different tasks (reading versus semantic judgment), direct contrasts indicated that abstract verbs generated greater activation in the left inferior frontal gyrus and temporal regions (middle temporal gyrus or posterior-lateral temporal areas), while concrete verbs lead to greater activation in more posterior temporal regions. The authors of these studies suggested that abstract verbs engage semantic processes more strongly in comparison to concrete verbs. To the best of our knowledge, the cognitive and neural correlates of concrete and abstract verb processing have not been investigated and compared in post-stroke aphasia – and thus this was a key target for the current study.

We also considered the effect of word frequency as it has been widely implicated in healthy adult language processing (e.g., Balota and Chumbley, 1984) and in some studies on aphasia (e.g., Cuetos et al., 2002, Nickels and Howard, 1995). Other aphasiological studies, particularly those on semantic aphasia, however, have shown an absent or reversed frequency effect (e.g., Hoffman et al., 2011a, Hoffman et al., 2011b). Again, the evidence to date has been mainly based on noun processing. Those studies that have explored verbs have shown an absent of frequency effect in word retrieval or sentence production tasks among patients with brain-injury (Kemmerer and Tranel, 2000), and aphasia (Bastiaanse et al., 2009, Bastiaanse et al., 2016). This might indicate that word frequency is not a factor that affects verb processing.

A review of the available neuropsychological and aphasiological assessment batteries suggests that there is a dearth of comprehensive tests to assess verb comprehension. While some tests have been specifically designed to assess verb deficits, they either tackle production but not comprehension, such as the Object and Action Naming Battery (OANB: Druks and Masterson, 2000), or they focus on syntactic impairments and sentence processing, such as the Northwestern Assessment of Verbs and Sentences (Thompson, 2011).

In the current study, a new neuropsychological test battery was developed to probe the semantic comprehension of verbs. This battery includes a synonym judgment test and a picture-to-word matching test. These tests are relatively challenging, leading to sensitive assessment of verb comprehension at single-word level. From a clinical perspective, this battery offers a new and important supplement to the existing clinical assessment tools. In particular, the abstract conditions of the synonym judgment test have the potential to detect mild comprehension deficits in cases that usually pass the typical (noun-based) clinical assessments but report comprehension deficits at the level of everyday functional communication (such as conversations or reading complicated notes, as insurance letters). Thus the key aims of this study were to investigate differences in the comprehension of concrete and abstract verbs for a large cohort of patients with chronic post stroke-aphasia, and further identify the neural correlates associated with verb processing using lesion-symptom mapping.

## 2 Methods

### 2.1 Constructing the battery

**Fig. 1:**
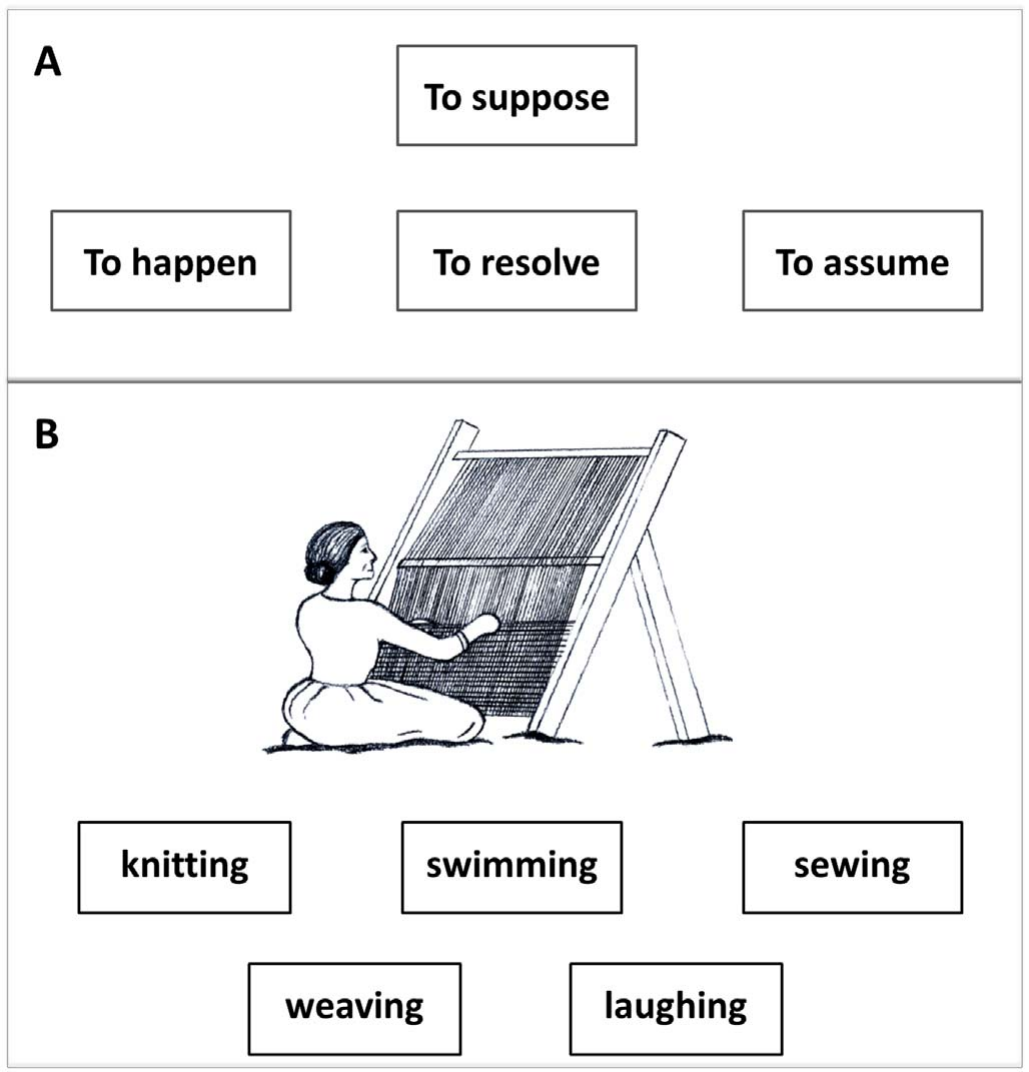
An illustration of the stimuli presented to the patients. A: example stimulus from the verb synonym judgment test: probe ‘To suppose’, target ‘To assume’, and distractors ‘To happen’ and ‘To resolve’. B: example stimulus from the verb picture-to-word matching test: this is an illustration picture, the actual picture used was item number 97 from the action pictures (OANB: Druks and Masterson, 2000), target response ‘weaving’, semantic distractors ‘sewing’ and ‘knitting’, and unrelated distractors ‘swimming’ and ‘laughing’.

Two novel neuropsychological tests were developed to examine single-word concrete and abstract verb comprehension. First, a verb synonym judgment test was constructed. This test consists of 80 verb stimuli split evenly into four conditions: concrete high-frequency verbs (e.g., ‘to park’), concrete low-frequency verbs (e.g., ‘to bandage’), abstract high-frequency verbs (e.g., ‘to suppose’), and abstract low-frequency verbs (e.g., ‘to cogitate’). Two methods are commonly implemented to categorise words into concrete/abstract words: (1) subjectively rate the concreteness of the word based on the degree to which a word relates to a tangible entity, or (2) subjectively rate the imageability of the word (Hoffman, 2015, Paivio et al., 1968). It has been shown that these two constructs are highly correlated (*r* = 0.83: Paivio et al., 1968), and therefore, most studies treat concreteness and imageability as interchangeable and use either construct to distinguish concrete from abstract words. In this study, we used imageability ratings to distinguish concrete and abstract verbs. All verb probes and imageability ratings were drawn from a corpus of published norms for verbs (Bird et al., 2001a). Log frequency values (logarithm of combined written and spoken count divided by total words in corpus) for verbs taken from the CELEX Database (Baayen et al., 1995) and the British National Corpus (Consortium, 2007) were also manipulated for all verbs in the test. Each trial consists of a probe that was presented alongside three written words choices: one semantically related (target) verb, and two unrelated verb distractors. An example trial is illustrated in Fig. 1A. All probes within the same conditions were carefully matched on their imageability and frequency values, and within each trial, the target synonym was matched to the probe on word frequency and both distractors were matched to the probe on imageability and frequency. Table 1 summarises the imageability and frequency values for each verb condition.

**Table 1:** Mean (and SD) of imageability and frequency values for each condition in the synonym judgment test.

**Verb condition**	**Probe**		**Target**	**Distractor 1**		**Distractor 2**	
	Imageability^a^	Frequency^b^	Frequency	Imageability	Frequency	Imageability	Frequency
Concrete high-frequency	5.38 (0.25)	3.56 (0.47)	3.44 (0.70)	5.35 (0.20)	3.49 (0.52)	5.30 (0.56)	3.52 (0.43)
Concrete low-frequency	5.34 (0.27)	2.02 (0.43)	2.19 (0.49)	5.38 (0.31)	2.02 (0.42)	5.39 (0.53)	2.03 (0.73)
Abstract high-frequency	2.99 (0.25)	3.62 (0.67)	3.76 (0.46)	2.93 (0.37)	3.77 (0.96)	3.22 (0.55)	3.71 (0.48)
Abstract low-frequency	2.94 (0.33)	2.07 (0.50)	2.39 (0.58)	2.78 (0.40)	2.00 (0.60)	2.76 (0.34)	1.96 (0.64)

Note: all comparisons between the choice (target or distractor) group and the probe group using independent *t*-tests are not significantly different (2-tailed *p*-values > 0.1).

Verbs in the concrete conditions are with imageability values above 5 on a 7-point rating scale, and the ones in the abstract conditions are with values below 3.3. Median split was not used to avoid including verbs with medium imageability.

a Matched imageability ratings of the two concrete conditions (*t* = 0.48, *p* = 0.63) and the two abstract conditions (*t* = 0.58, *p* = 0.56).

b Matched frequency ratings of the two high-frequency conditions (*t* = 0.36, *p* = 0.72) and the two low-frequency conditions (*t* = 0.12, *p* = 0.9).

Second, a verb picture-to-word matching comprehension test was developed in order to examine whether there are differences in performance between comprehension and production of verbs. Examining the production of abstract verbs is highly challenging because abstract items cannot be easily represented in pictorial form. An alternative could be to use content analyses of connected speech rather than single word tasks, although this can be very time-consuming. Therefore, the focus of this part of the study (comparison between verb production and comprehension) was on concrete verbs. In order to avoid an overlap of the concrete verbs between the first and the second tests, this picture-to-word matching comprehension test was constructed using a different set of verbs: all 100 verbs from the OANB (Druks and Masterson, 2000). The OANB is a widely used naming test in research and in clinical practice, and the construction of a new comprehension test that is based on this naming test, allows direct comparison between performance across the two modalities (comprehension and production). In the newly developed picture-to-word matching test, the picture was presented with five multiple-choice printed words, including the target response (e.g., ‘weaving’), two semantically related distractors (e.g., ‘knitting’ and ‘sewing’) and two unrelated distractors (e.g., ‘swimming’ and ‘laughing’). An example is illustrated in Fig. 1B. All verbs were viewed in the present continuous tense to avoid confusion with nouns (e.g., ‘combing’).

Both tests were piloted among eight English speaking healthy younger adults. Results on the synonym judgment test showed 93.26% accuracy. It was identified that the incorrect responses related to noun-verb ambiguous words (e.g., ‘butter’ and ‘fence’). Therefore, all verbs in the test were changed to be viewed in their infinitive form preceded with the particle ‘to’ (e.g., ‘to butter’ and ‘to fence’), in order to disambiguate any noun-verb ambiguous words. Additionally, one verb ‘fetter’ received 50% item accuracy and thus it was replaced with a verb from the same category (abstract low frequency). The results on the picture-to-word matching test showed 99.5% accuracy. Twenty-eight semantic distractors were identified by at least 25% of the participants as potential correct response (e.g., ‘stacking’ for ‘building’, ‘shovelling’ for ‘digging’). Therefore, they were replaced with different semantic distractors, and subsequently the test was piloted again with 100% accuracy and no issues with the distractors. A list of the items constructing the verb synonym judgment test and the picture-to-word matching test is available in Supplementary Appendix A.

### 2.2 Normative data

Normative data on the newly developed tests were collected from twenty-five (9 males and 16 females) non-brain-damaged elderly control participants. All were native English-speakers, right handed, aged between 61 and 86 (mean = 71.64, SD = 5.37), and were in formal education between 10 to 19 years (mean = 14.4, SD = 3.1). All participants reported no history of any neurological condition or brain-injury, and their scores on the Mental State Examination (Folstein et al., 1975) were above 26 (mean = 28.92, SD = 1.07). Both group accuracy and items accuracy were examined on both tests.

The group accuracy results revealed ceiling performance on both tests; the mean group accuracy on the synonym judgment test was 78.92 (SD = 1.28, 98.65%), and the picture-to-word matching test was 99.52 (SD = 0.9, 99.52%). Furthermore, responses to each verb condition in the verb synonym judgment test (20 items in each category) indicated celling performance in all four conditions. The concrete high-frequency condition obtained the highest accuracy (mean = 19.96, SD = 0.2), followed by concrete low-frequency condition (mean = 19.88, SD = 0.32), and abstract high-frequency condition (mean = 19.56, SD = 0.75), and lastly the abstract low-frequency condition (mean = 19.52, SD = 0.90). There was no correlation between control participants' scores on both tests with their age or education.

A detailed examination of the responses obtained for each item within both tests was high and, therefore, there was no need to replace or remove any items: (i) synonym judgment test: all concrete high-frequency verbs obtained an accuracy above 96%, and all verbs in the other three conditions obtained an accuracy above 92%. The most incorrectly identified verb was from the abstract low-frequency condition, ‘to hone’, which obtained 88% accuracy; (ii) picture-to-word matching test: 90 verbs obtained 100% consistent correct responses, nine verbs obtained 96%, and one verbs ‘drawing’ obtained 88%. As the vast majority of control participants (*N* = 22) identified the verbs with 88% correctly, these were included in the tests.

### 2.3 Participants with aphasia

Forty-eight patients who had developed aphasia following a single left haemorrhagic or ischaemic stroke participated in this study (34 males and 14 females). The Boston Diagnostic Aphasia Examination (BDAE: Goodglass and Kaplan, 1983) was administered to each participant, and their aphasia was classified using the BDAE standard aphasia classification criteria (for details on the BDAE language profiles and definition of each classification, please see Goodglass and Kaplan, 1972). All participants were at least 12 months post-stroke at the time of scanning and testing, and were native English speakers with normal or corrected-to-normal vision and/or hearing. Their age ranged between 44 and 87 (mean = 63.31, SD = 11.8), and their education varied from 9 to 19 years (mean = 12.58, SD = 2.5). The exclusion criteria included more than one stroke or any other neurological conditions, any contraindications for MRI scanning, and being pre-morbidly left-handed. No restrictions were placed according to aphasia severity or classification, in order to sample the full range of severity and classifications of aphasia. Demographic information is presented in Table 2. Informed consent was obtained from all participants prior to participation under approval from local ethics committee.

**Table 2:** Participants demographic information arranged according to their lesion volume.

**Participant ID**	**Age** (years)	**Gender**	**Education** (years)	**BDAE aphasia classification**	**Time-post onset** (months)	**Lesion volume** (voxels 2 mm^3^)
112	44	Female	16	Anomic	21	175
123	54	Female	11	Anomic	65	1526
130	70	Male	11	Anomic	40	3311
128	47	Female	16	Conduction	34	3897
146	49	Male	19	Anomic	37	4538
131	69	Male	11	Conduction	49	4773
116	67	Male	11	Conduction	17	4879
124	49	Female	13	Anomic	62	5273
109	64	Male	12	Transcortical sensory	71	5822
140	66	Male	17	Conduction	16	6557
145	65	Male	10	Anomic	85	6607
134	56	Male	16	Anomic	18	6974
110	46	Female	11	Anomic	81	6975
147	85	Male	9	Anomic	48	7854
104	69	Female	19	Anomic	37	8118
120	46	Male	11	Anomic	62	8437
114	87	Male	9	Anomic	21	8528
144	70	Male	11	Anomic	77	8788
125	71	Female	19	Anomic	56	9159
133	54	Female	12	Anomic	129	9767
107	45	Male	14	Anomic	27	10,409
118	53	Male	17	Broca's	100	11,915
139	75	Female	11	Mixed non-fluent	168	12,057
108	83	Male	9	Broca's	24	12,131
102	58	Female	15	Anomic	280	12,699
135	60	Male	13	Broca's	58	13,080
122	77	Female	11	Broca's	74	13,577
143	60	Male	13	Mixed non-fluent	76	14,625
103	60	Male	11	Anomic	49	16,433
117	82	Male	12	Broca's	122	18,163
101	59	Male	15	Broca's	184	18,392
136	55	Male	13	Broca's	56	18,632
105	44	Female	11	Anomic	32	18,948
111	74	Male	11	Global	24	19,500
127	74	Male	11	Mixed non-fluent	42	22,732
137	52	Male	13	Anomic	90	22,948
106	73	Female	12	Transcortical mixed	54	23,863
126	52	Male	12	Broca's	73	26,218
141	68	Male	11	Mixed non-fluent	53	31,317
142	65	Male	12	Mixed non-fluent	102	31,599
121	64	Male	11	Mixed non-fluent	36	33,239
129	58	Male	13	Global	74	33,239
148	80	Male	11	Mixed non-fluent	64	33,678
138	79	Male	13	Mixed non-fluent	56	34,242
115	74	Male	11	Broca's	117	36,877
113	63	Male	11	Global	76	37,822
132	54	Female	11	Mixed non-fluent	118	40,313
119	70	Male	12	Global	68	41,379

### 2.4 Procedure

The two newly-developed comprehension tests and a naming test were administrated in one testing session; the naming test was first followed by the synonym judgment test, and then the picture-to-word matching. After the administration of each test, a break was taken followed by an administration of another test (not included in this study). The naming test included 98 action pictures from the OANB (this includes items that obtained high name agreement > 88% from the control participants), and this was administrated before the picture-to-word matching test, in order to avoid any cueing effects on the naming scores. All three tests were administered following an example item, and three to five practice items that were not included in the main test, to ensure that participants understood the task. Items were presented separately on a laptop screen in a randomised order using E-prime® (Psychology Software Tools Inc., Sharpsberg, Philadelphia). In the synonym judgment and picture-to-word matching tests, the probe was presented in the top part of the screen and the target and distractors were displayed underneath. Simultaneous auditory and visual presentation for all verbs was used and neither test was timed. In the naming test, each picture was presented separately on the screen for 10 s, and participants were instructed to name what is happening in the picture, or what is the person in the picture doing?. The initial verb response was entered in the accuracy analysis and the use of an inflectional form of the verb was not considered in the scoring (i.e. any form of the verb was accepted).

### 2.5 Acquisition and processing of neuroimaging data

High-resolution structural T1-weighted MRI scans were acquired for each participant on a 3.0 T Philips Achieva scanner (Philips Healthcare, Best, The Netherlands) using an eight-element SENSE head coil. A T1-weighted inversion recovery sequence with 3D acquisition was utilised, with the following parameters: repetition time = 9.0 millisecond (ms), echo time = 3.93 ms, acquired voxel size = 1.0 × 1.0 × 1.0 mm^3^, slice thickness = 1 mm, matrix size = 256_256, 150 contiguous slices, flip angle = 8, field of view = 256 mm, inversion time = 1150 ms, SENSE acceleration factor 2.5, total scan acquisition time = 575 s.

**Fig. 2:**
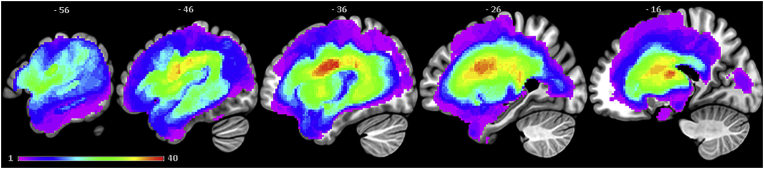
Lesion overlap map across 48 post-stroke aphasia patients illustrating the distribution of lesions. Colour scale indicates number of patients with a lesion at that location. Image threshold = 1–40. The maximum number of participants who had a lesion in one voxel was 40 (MNI coordinate: − 38, − 9, 24; central opercular cortex). (For interpretation of the references to colour in this figure legend, the reader is referred to the web version of this article.)

Participants' structural T1-weighted MRI scans were pre-processed with Statistical Parametric Mapping software (SPM8: Wellcome Trust Centre for Neuroimaging, http://www.fil.ion.ucl.ac.uk/spm/) running under Matlab 2012a. Before performing the segmentation and normalisation, we stripped non-brain tissue from the T1 images using an optimised brain extraction tool for lesioned brains (OptiBET: Lutkenhoff et al., 2014). The resultant images were then normalised into standard Montreal Neurological Institute (MNI) space using a modified unified segmentation-normalisation procedure optimised for focal brain lesions (Seghier et al., 2008). Structural imaging scans from a healthy age and education matched control group (18 male and 4 female; mean age = 69.13 years, SD = 5.85, range = 59–80; and mean education = 13 years, SD = 2.66, range = 10–18) were used as a reference to identify lesion/abnormal tissue in the stroke patients. Structural MRI scans from these 22 healthy controls and all 48 patients with post-stroke aphasia were entered into the segmentation-normalisation procedure. This procedure combines segmentation, spatial normalisation and bias correction through the inversion of a single unified model, which combines tissue classes (grey and white matter, cerebral spinal fluid (CSF), and an additional tissue class for abnormal voxels), intensity bias and non-linear warping into the same probabilistic models that are assumed to generate individual-specific brain images (details available in Ashburner and Friston, 2005). This procedure essentially detects areas of neural abnormality in an unexpected tissue class, and therefore, identifies missing grey and white matter as well as areas of augmented CSF space. Each patient's lesion was therefore automatically identified using this fully automated method based on fuzzy clustering (Seghier et al., 2008). The default parameters in the automated lesion identification toolkit were used aside from the lesion definition ‘U-threshold’, which was set to 0.5 rather than 0.3 to create a binary lesion image. This modification was done after comparing the results obtained from a sample of patients to what would be nominated as lesioned tissue by an expert neurologist. Images were then smoothed with an 8 mm full-width-half-maximum Gaussian kernel, in order to account for the global intra-individual shape differences, and were then used in the lesion-symptom mapping analyses. The images generated for each patient were visually inspected with respect to the original scan and were then used to generate a lesion overlap map (Fig. 2), which primarily covers the left hemisphere area supplied by middle cerebral artery (MCA) (Phan et al., 2005).

### 2.6 Analyses of neuroimaging data

In order to identify the neural correlates associated with semantic verb processing, patients' individual behavioural scores were correlated with the normalised-smoothed T1-weighted images across the whole brain, using a Voxel-Based Correlational Methodology (VBCM: Tyler et al., 2005) conducted in SPM8 and running under Matlab 2012a. This method is a variant of voxel-based lesion symptom mapping (VLSM: Bates et al., 2003), but instead of using a binary classification for brain tissue (intact versus lesioned), a continuous measure of signal intensity is used and correlated with behavioural data, in order to preserve the continuous nature of both neural and behavioural patterns. Several VBCM analyses were conducted in this study. Firstly, behavioural scores from the synonym judgment test were correlated with tissue intensity to identify the neural correlates of single-word semantic verb comprehension. Secondly, to investigate whether performance on verb synonym judgment was mediated by word concreteness and to identify the neural correlates associated with concrete and abstract verbs, behavioural scores from the concrete and abstract verbs were correlated with tissue intensity in separate models, and in a direct contrast between them. Thirdly, the neural correlates of high and low-frequency verbs were explored separately, and also directly contrasted in order to understand subtle differences within the synonym judgment test. Finally, the neural correlates for verb naming and verb picture-to-word matching were compared by looking at the lesion correlates in separate models as well as directly contrasting them. All VBCM analyses were carried out using multiple regression models on normalised-smoothed T1-weighted images with test scores entered as regressors and the results were thresholded at *p* < 0.0005 voxel-level and cluster corrected using family-wise error (FWE) of *p* < 0.05. This stringent threshold was used rather than the standard threshold, in order to increase the specificity of the large clusters associated with each condition when they were examined in a separate models. For all direct contrasts, scores on both behavioural conditions were entered simultaneously in the same model and the standard threshold was used at *p* < 0.001 voxel-level and cluster corrected using FWE of *p* < 0.05, as the effects are expected to be more subtle. Subsequently, all VBCM analyses were repeated with three demographic variables (age, education, and time since stroke onset) entered in the same model as covariates. Moreover, each patient's lesion volume (proxy of neurological severity) obtained from the output of the automated lesion identification procedure (Seghier et al., 2008) was entered as a covariate in subsequent VBCM analyses. It is important to note, however, that by partialling out some covariates and especially lesion volume there is a high risk for type II error. Hence, all VBCM analyses were performed and reported in this paper once with the behaviours of interest only, then with demographic variables entered as covariates, and finally with a correction for lesion volume. This protocol was followed in order to account for both type I and type II errors. The analysis conducted with covariate were at the standard threshold of *p* < 0.001 voxel-level and cluster corrected using FWE *p* < 0.05. The neural correlates were described using the Harvard-Oxford atlas in MNI space (Desikan et al., 2006), and natbrainlab white matter atlas based on diffusion tensor tractography (Catani et al., 2012). The figures were produced using the MRIcron software (Rorden et al., 2007).

## 3 Results

### 3.1 Validity and reliability of the developed test battery

To examine the validity of the verb synonym judgment and the verb picture-to-word matching tests, patients' score on these two tests were compared, using Pearson's correlation, to their scores obtained on five independent semantic comprehension tests: spoken and written word-to-picture matching tests (Bozeat et al., 2000), 96 trial synonym judgment test (Jefferies et al., 2009), Camel and Cactus test (Bozeat et al., 2000), and spoken sentence comprehension task from the Comprehensive Aphasia Test (CAT: Swinburn et al., 2005). The results revealed significant positive correlations between all tests (all *r* > 0.71, *p* < 0.0001), indicating high validity for both tests. The pair-wise correlations are illustrated in Table 3.

**Table 3:** Correlations between the newly developed tests and other semantic comprehension tests.

	**Verb synonym judgment**^a^	**Verb picture-to-word matching**^a^	**Spoken word-to-picture matching**	**Written word-to-picture matching**	**Camel and cactus**	**96 trial synonym judgment**	**Spoken sentence comprehension**
Verb synonym judgment^a^	1.00	0.87	0.71	0.74	0.83	0.9	0.81
Verb picture-to-word matching^a^	–	1.00	0.88	0.89	0.92	0.94	0.78

All correlations are significant at *p* < 0.0001.

a Tests developed in this study.

The reliability of the two tests was examined using the split-half method, in which each test is split into two halves and the patients' scores are compared using Pearson's correlation. The result revealed strong positive correlation between the two halves on the verb picture-to-word matching test (*r* = 0.97, *p* < 0.0001), and the synonym judgment (*r* = 0.99, *p* < 0.0001). These results indicate high internal reliability on both tests.

### 3.2 Behavioural results on participants with aphasia

#### 3.2.1 Processing concrete and abstract verbs

Patients with aphasia performed significantly lower than the control group on the verb synonym judgment test (*t* = 7.6, two-tailed *p* < 0.0001).

**Fig. 3:**
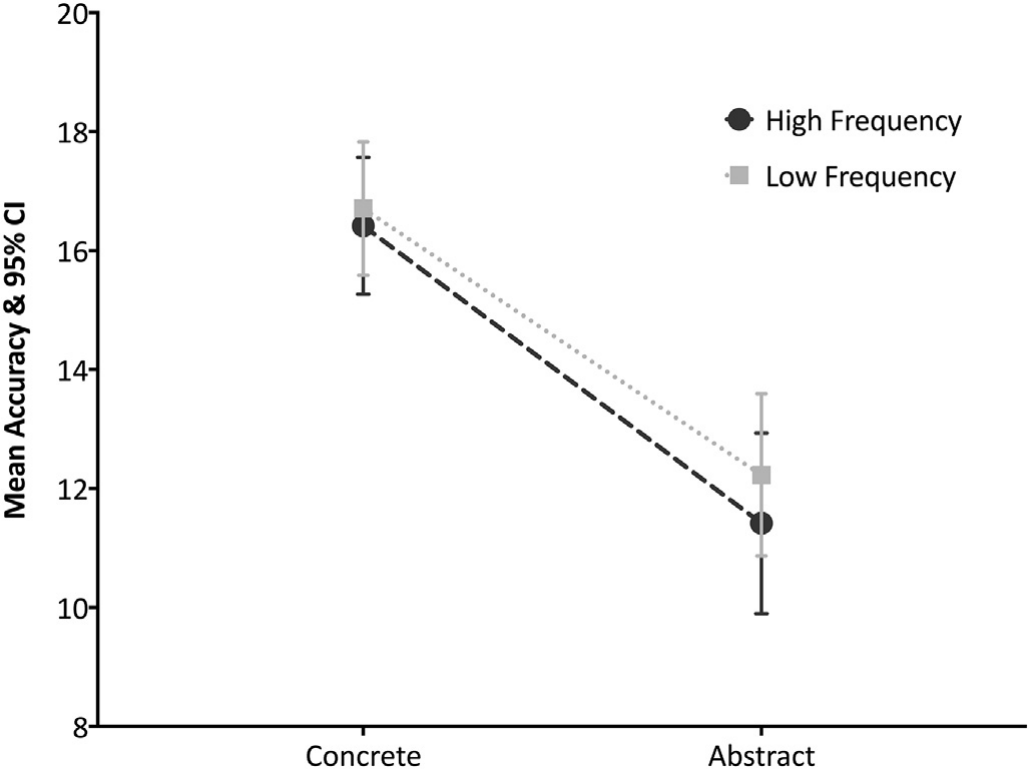
Performance on the verb synonym judgment test. 2 × 2 repeated measure ANOVA showing an effect of imageability, with an advantage of concrete over abstract verbs, with no frequency or interaction effects on verb processing. Error bars show the standard error of mean correct responses.

The average performance from the aphasia group on the synonym judgment test was 70.9% (mean = 56.78, SD = 15.7). The highest scores were obtained in response to the concrete low-frequency (mean = 16.7, SD = 3.86) and the concrete high-frequency verbs (mean = 16.4, SD = 3.96), followed by the abstract low-frequency (mean = 12.22, SD = 4.7), and the abstract high-frequency verbs (mean = 11.42, SD = 5.22). A 2 × 2 repeated measure ANOVA was performed to examine the effect of imageability, frequency and their interaction on verb comprehension (Fig. 3). This analysis revealed a significant effect of imageability (F(1,47) = 128.53, *p* < 0.0001, η^2^ = 0.73), with higher scores in response to concrete verbs (mean = 33.125, SD = 7.5) compared to abstract verbs (mean = 23.64, SD = 8.6). The results did not identify a significant effect of frequency: responses to high-frequency verbs (mean = 27.83, SD = 8.58) did not significantly differ from responses to low-frequency verbs (mean = 28.93, SD = 7.7). The interaction effect between imageability and frequency on verb processing was also not significant. The effect of imageability remained significant (*p* < 0.0001) even when aphasia severity (as measured by the BDAE aphasia severity rating scale) was included as a covariate in this ANOVA, despite the significant correlation between participants' aphasia severity and their performance on the synonym judgment test (*r* = 0.73, *p* < 0.0001). Descriptive statistics are illustrated in Table 4. Additionally, by inspecting individual performances, it has been observed that all participants performed better in response to concrete verbs compared to abstract ones.

#### 3.2.2 Verb production and comprehension

Patients with aphasia performed significantly lower than the control group on the verb picture-to-word matching test (*t* = 4.37, two-tailed *p* < 0.0001).

Patients with aphasia performed better on the verb picture-to-word matching test (mean = 85.17, SD = 12.26, 86.9%) compared to the corresponding items from the naming test (mean = 51.08, SD = 31.47, 53.22%) that was based on 98 verbs from the OANB (*t*(47) = 10.28, two-tailed *p* < 0.0001). This difference remained significant (*p* < 0.0001) after accounting for aphasia severity, which was significantly correlated with participants' performance on the verb picture-to-word matching test (*r* = 0.78, *p* < 0.0001), and the naming test (*r* = 0.83, *p* < 0.0001). Descriptive statistics are illustrated in Table 4. All individual participants performed with higher accuracy on the verb comprehension test compared to the verb naming test.

**Table 4:** Descriptive statistics of the behavioural results.

**Accuracy**	**Naming** (max = 98)	**Picture-to-word matching** (max = 98)	**Verb synonym judgment** (max = 80)	**Concrete high-frequency** (max = 20)	**Concrete low-frequency** (max = 20)	**Abstract high-frequency** (max = 20)	**Abstract low-frequency** (max = 20)
Mean (SD)	51.08 (31.47)	85.16 (12.26)	56.77 (15.75)	16.42 (3.97)	16.71 (3.86)	11.42 (5.23)	12.23 (4.7)

The lexical effect of verb argument structure has been widely addressed in the literature on verb production in aphasia (e.g., Cho-Reyes and Thompson, 2012), and therefore this has been examined in this study. The verbs used in the picture-to-word matching and naming tests were classified into one-argument verbs (*N* = 43), and two-argument verbs (*N* = 55). The method used to classify the verbs was based on the number of arguments the verb takes in the pictorial image: if the picture illustrates an agent only (e.g., ‘barking’) then the verb was classified as a one-argument verb, whereas if the picture illustrates an agent and a patient (e.g., ‘stroking’) then the verb was classified as a two-argument verb. This method was used to overcome the challenge of classifying some optional one or two-argument verbs (e.g., ‘driving’). The comparisons between the two types of verb argument structure were carried out on percentage accuracy due to the different number of items in the two groups. The results revealed no significant naming difference between producing one-argument verbs (mean = 52.32, SD = 32.8) and two-argument verbs (mean = 51.74 SD = 32.54). Additionally, there was no significant difference in the comprehension of one-argument verbs (mean = 87.16 SD = 12.05) and two-argument verbs (mean = 86.67 SD = 13.49) on the picture-to-word matching test. These results indicate a lack of argument structure effect on both verb naming and comprehension.

### 3.3 Neuroimaging results on participants with aphasia

#### 3.3.1 Neural correlates of verb semantic comprehension

**Fig. 4:**
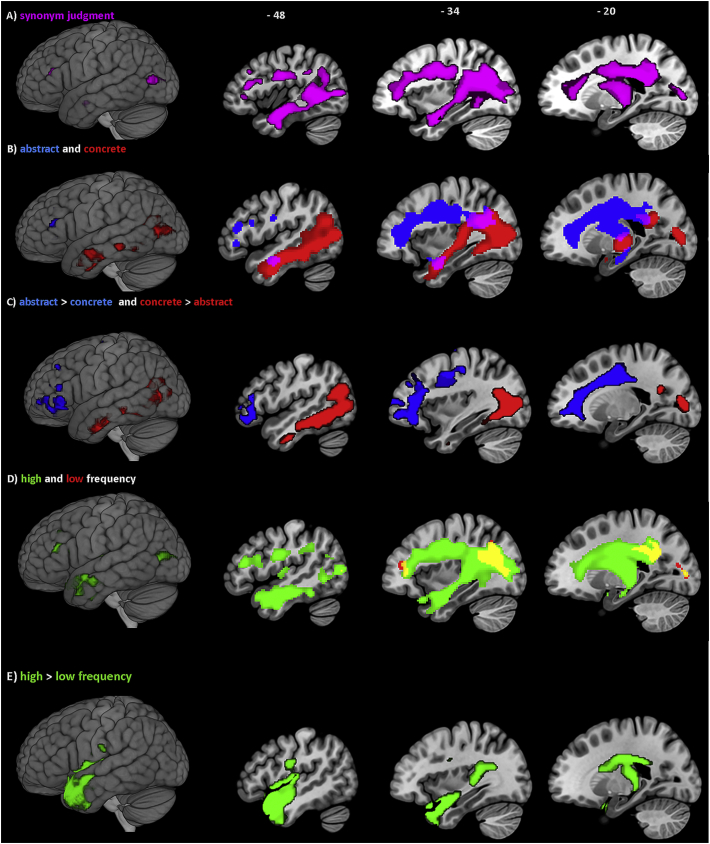
Lesion-symptom mapping showing neural correlates associated with single-word semantic verb comprehension. Results threshold at *p* < 0.0005 voxel-level and FWE cluster corrected *p* < 0.05, unless stated otherwise: (A) Verb synonym judgment test. (B) Abstract (blue) and concrete (red) verbs, and overlapping regions (violet). (C) Direct contrast between processing abstract (blue) and concrete verbs (red), thresholded at *p* < 0.001 voxel-level and FWE cluster-level corrected *p* < 0.05. (D) High (green) and low (red) frequency verbs, and overlapping regions (yellow). (E) Direct contrast between processing high (green) and concrete verbs, thresholded at *p* < 0.001 voxel-level and FWE cluster-level corrected *p* < 0.05. (For interpretation of the references to colour in this figure legend, the reader is referred to the web version of this article.)

The first VBCM analysis shows the neural correlates associated with verb comprehension during synonym judgment test (Fig. 4A). The identified neural regions included the left superior and inferior lateral occipital cortex, anterior and posterior middle temporal gyrus, anterior inferior temporal gyrus, temporal pole, planum polare, superior parietal lobule, angular gyrus, pre-central gyrus, inferior and middle frontal gyrus and frontal pole. This cluster also encompassed the left posterior cingulate cortex, thalamus and fornix and white matter tracts corresponding to the left inferior longitudinal fasciculus, posterior segment of arcuate fasciculus and inferior occipito-frontal fasciculus. Similar results were obtained when age, education and time post onset were added to this analysis as covariates. This cluster, however, dropped out of significance when lesion volume was added to the analysis as a covariate.

The second VBCM analysis identified the neural correlates associated with concrete and abstract verb processing (Fig. 4B). This indicated common regions associated with processing both concrete and abstract verbs (Dice similarity coefficient = 0.18) in the left anterior temporal gyrus, posterior supramarginal gyrus and superior lateral occipital cortex. The neural correlates for concrete verbs extended into the left anterior and posterior temporal fusiform cortex, anterior and posterior inferior temporal gyrus, posterior middle temporal gyrus, planum polare, Heschl's gyrus (H1 and H2), angular gyrus, and superior and inferior lateral occipital cortex. This cluster also encompassed the precuneus and white matter tracts corresponding to the anterior inferior longitudinal fasciculus, posterior segment of arcuate fasciculus, and inferior occipito-frontal fasciculus. The neural correlates associated with abstract verb processing extended into the left frontal pole, inferior frontal gyrus, pre-central gyrus, orbito-frontal gyrus, middle frontal gyrus, and superior parietal lobule. This cluster also encompassed the posterior cingulate cortex, internal capsule and white matter tracts corresponding to the superior and inferior longitudinal fasciculus, inferior occipito-frontal fasciculus, anterior and posterior segment of arcuate fasciculus and cingulum. Similar results were obtained when age, education and time post onset were added to the analysis as covariates. The identified clusters associated with concrete verb processing were powerful enough to survive the analysis when lesion volume was added as a covariate; clusters associated with abstract verbs, however, did not survive correction for lesion volume.

The direct contrast between concrete and abstract verbs (Fig. 4C) revealed that left frontal regions including the inferior frontal gyrus, frontal pole, pre-central gyrus, orbito-frontal gyrus, middle frontal gyrus, and white matter tracts corresponding to the superior inferior longitudinal fasciculus and cingulum were associated with abstract verb processing over-and-above concrete verb processing. On the other hand, left posterior temporal and occipital regions were associated with concrete verb processing over-and-above abstract verb processing; these regions include posterior middle temporal gyrus, posterior and anterior inferior temporal gyrus, angular gyrus, and superior and inferior lateral occipital cortex. This cluster also encompassed the precuneus, internal capsule and white matter tracts corresponding to the inferior longitudinal fasciculus and posterior segment of arcuate fasciculus. When age, education and time post onset were added to the analysis as covariates; similar results were obtained for the concrete over abstract verb processing; whereas, the cluster associated with the abstract over concrete verb processing was similar except that it did not include the pre-central gyrus and cingulum. The identified clusters associated with concrete over abstract verb processing were powerful enough to survive the analysis when lesion volume was added as a covariate; clusters associated with abstract verbs, however, did not survive correction for lesion volume. The distinct patterns of neural correlates associated with concrete and abstract verb processing indicate that the neural correlates associated with verb synonym judgment are mediated by word concreteness.

The third VBCM analysis explored the neural correlates associated with high and low-frequency verbs and showed overlapping regions (Dice similarity coefficient = 0.21) in the left frontal pole, superior parietal lobule, and superior and inferior lateral occipital cortex (Fig. 4D). Accuracy on high-frequency verbs further correlated with the left anterior and posterior middle temporal gyrus, anterior inferior temporal gyrus, temporal pole, planum polare, pre-central gyrus, angular gyrus, and white matter tracts corresponding to the posterior segment of the arcuate fasciculus, inferior longitudinal fascicules, and inferior occipito-frontal fasciculus. On the other hand, accuracy on low-frequency verbs further correlated with white matter tracts corresponding to the inferior longitudinal fasciculus, posterior segment of the arcuate fasciculus and the cingulum. Similar results were obtained when age, education and time post onset were added to the analysis as covariates. These clusters, however, dropped out of significance when lesion volume was added to this analysis as a covariate.

The results from the direct contrast (Fig. 4E) revealed a large cluster covering the left temporal pole, anterior superior and middle temporal gyrus, planum polare, pre- and post-central gyrus, insular cortex and central opercular cortex and white matter tracts corresponding to the left inferior longitudinal fasciculus associated with high-frequency over-and-above low-frequency verb processing. The direct contrast also revealed another cluster for high-frequency words, which could reflect patients with enlarged/abnormal ventricles rather than actual tissue damage; as it covered subcortical regions including caudate and thalamus, while extending to cover white matter tracts corresponding to uncinate, fornix and subcallosal cortex. Similar results were obtained when age, education and time post onset were added to this analysis as covariates, except that the cluster extended posteriorly to cover the left posterior supramarginal gyrus and parietal operculum cortex. These clusters, however, dropped out of significance when lesion volume was added to this analysis as a covariate. No clusters were associated with processing low-frequency over-and-above high-frequency verb, even at the lower threshold of *p* < 0.01 voxel-level, FWE cluster-level corrected at *p* < 0.05. Significant clusters and main peak MNI coordinates are listed in Table 5.

**Table 5:** Details of the significant clusters and peak MNI coordinates associated with single-word verb semantic comprehension based on the synonym judgment test. Anatomical labels obtained using the Harvard-Oxford and natbrainlabs atlases in MNI space.

**Test/condition**				**MNI co-ordinates**		
	**Location**	**Cluster size (voxels)**	**Z**	*x*	*y*	*z*
Verb synonym judgment	Superior lateral occipital cortex	11,829	4.91	− 28	− 76	12
	Posterior segment of arcuate fasciculus		4.61	− 34	− 52	22
	Posterior inferior longitudinal fasciculus		4.58	− 32	− 55	20
	Cortico-spinal tract		4.56	− 28	− 44	30
	Anterior inferior longitudinal fasciculus		4.49	− 38	− 8	− 20
	Frontal pole		4.45	− 28	40	16
	Posterior cingulate cortex		4.40	8	− 34	26
	Inferior lateral occipital cortex		4.26	− 46	− 80	8
	Posterior middle temporal gyrus		4.13	− 50	− 10	− 20
	Pre-central gyrus		4.09	− 38	− 14	32
	Anterior inferior temporal gyrus		4.08	− 44	− 10	− 32
	Fornix		3.64	− 18	− 20	14
	Thalamus		3.44	− 14	− 24	4
	Anterior middle temporal gyrus		3.41	− 50	− 6	− 26
	Inferior frontal gyrus		3.39	− 38	30	18
Concrete verbs	Superior lateral occipital cortex	11,445	6.43	− 28	− 82	8
	Inferior lateral occipital cortex		6.34	− 42	− 64	0
	Cortico-spinal tract		5.35	− 22	− 24	− 2
	Posterior inferior temporal gyrus		5.16	− 52	− 60	− 8
	Anterior inferior temporal gyrus		4.93	− 42	− 8	− 36
	Posterior middle temporal gyrus		4.76	− 58	− 14	− 22
	Anterior inferior longitudinal fasciculus		4.44	− 38	− 8	− 22
	Anterior middle temporal gyrus		3.99	− 50	− 6	− 26
	Posterior segment of arcuate fasciculus		3.88	− 38	− 44	20
Abstract verbs	Frontal pole	9298	4.60	− 28	40	16
	Anterior parahippocampal gyrus		4.47	− 14	− 16	− 24
	Cortico-spinal tract		4.43	− 26	− 42	30
	Cingulum		4.40	− 18	− 26	32
	Internal capsule		4.35	− 20	− 12	34
	Middle frontal gyrus		4.28	− 32	0	36
	Pre-central gyrus		4.25	− 36	− 14	32
	Inferior frontal gyrus		4.18	− 48	34	14
	Posterior cingulate cortex		3.90	4	− 30	26
	Anterior segment of arcuate fasciculus		3.36	− 38	− 24	24
	Posterior segment of arcuate fasciculus		3.34	− 38	− 44	20
	Inferior longitudinal fasciculus	621	4.23	− 38	− 8	− 20
Abstract > concrete verbs^a^	Middle frontal gyrus	2436	4.01	− 28	34	22
	Inferior frontal gyrus		4.01	− 28	36	10
	Frontal pole		3.85	− 28	46	6
	Orbito-frontal cortex		3.20	− 38	36	− 6
Concrete > abstract verbs^a^	Inferior lateral occipital cortex	3877	6.49	− 42	− 64	− 2
	Posterior inferior longitudinal fasciculus		5.60	− 28	− 82	6
	Posterior inferior temporal gyrus		4.83	− 52	− 58	− 8
	Anterior inferior temporal gyrus		4.26	− 54	− 10	− 32
	Posterior middle temporal gyrus		4.17	− 60	− 36	− 14
	Superior lateral occipital cortex		3.52	− 50	− 68	24
	Posterior middle temporal gyrus		3.30	− 50	− 24	− 16
	Posterior segment of arcuate fasciculus		3.28	− 50	− 48	− 4
High-frequency verbs	Posterior segment of arcuate fasciculus	19,330	5.03	− 34	− 52	22
	Inferior longitudinal fasciculus		4.93	− 38	− 8	− 20
	Cortico-spinal tract		4.93	− 28	− 44	28
	Posterior inferior longitudinal fasciculus		4.87	− 30	− 76	12
	Inferior lateral occipital cortex		4.79	− 48	− 78	8
	Superior lateral occipital cortex		4.76	− 32	− 66	22
	Anterior middle temporal gyrus		4.53	− 52	− 8	− 20
	Posterior middle temporal gyrus		4.51	− 44	− 60	0
	Posterior segment of arcuate fasciculus		4.40	− 40	− 46	18
	Inferior frontal gyrus		3.72	− 40	14	22
	Temporal pole		3.07	− 40	14	− 34
Low-frequency verbs	Superior lateral occipital cortex	2044	4.69	− 26	− 76	14
	Cortico-spinal tract		3.88	− 28	− 46	30
	Inferior lateral occipital cortex		3.73	− 22	− 88	4
	Posterior segment of arcuate fasciculus		3.40	− 36	− 46	20
	Cingulum		3.35	− 18	− 48	42
	Frontal pole	398	4.17	− 30	42	16
High-frequency > low-frequency verbs^a^	Temporal pole	4053	5.01	− 36	16	− 26
	Insular cortex		4.66	− 40	0	− 16
	Uncinate		4.25	− 34	8	− 22
	Anterior middle temporal gyrus		4.03	− 60	2	− 14
	Central opercular cortex		3.88	− 48	− 10	6
	Inferior longitudinal fasciculus		3.56	− 46	2	− 24
	Anterior superior temporal gyrus		3.56	− 54	− 4	− 20
	Cortico-spinal tract	3553	3.79	− 28	− 30	10
	Caudate		3.62	− 10	0	14
	Suballosal cortex		3.60	− 4	12	− 14
	Fornix		3.55	− 18	− 20	14
	Thalamus		3.54	− 14	− 30	2

Results threshold at *p* < 0.0005 voxel-level, FWE cluster-level correction *p* < 0.05.

a Direct contrast at *p* < 0.001 voxel-level, FWE cluster-level correction *p* < 0.05.

#### 3.3.2 Neural correlates of verb production and comprehension

**Fig. 5:**
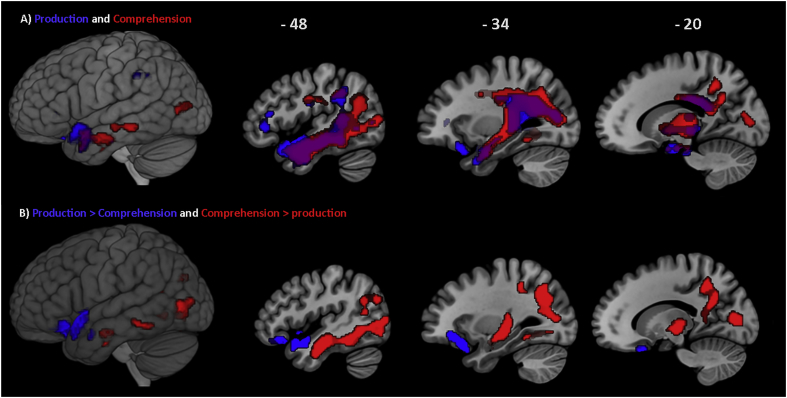
Lesion-symptom mapping analysis showing: (A) Neural correlates associated with concrete verb naming (blue) and comprehension (red), and their overlapping regions (purple), thresholded at *p* < 0.0005 voxel-level, and FWE cluster-level correction at *p* < 0.05. (B) A direct contrast showing the neural correlates associated with concrete verb naming (red) and comprehension (blue), thresholded at *p* < 0.01 voxel-level, and FWE cluster-level correction of *p* < 0.05. (For interpretation of the references to colour in this figure legend, the reader is referred to the web version of this article.)

The neural correlates identified from the VBCM analyses that were associated with production and comprehension of concrete verbs are shown in Fig. 5A. The results revealed a wide range overlapping regions associated performance with both tasks (Dice similarity coefficient = 0.61). This overlap comprised of the left temporal pole, anterior and posterior middle and inferior temporal gyri, posterior temporal fusiform cortex, planum polare, and internal capsule. Performance on concrete verb naming additionally correlated with lesions in the anterior superior temporal gyrus, anterior temporal fusiform cortex, and posterior supramarginal gyrus. This cluster also encompassed white matter tracts corresponding to the left anterior and posterior segment of the arcuate fasciculus, inferior longitudinal fasciculus and inferior occipito-frontal fasciculus. Performance on the verb naming was also correlated with a second frontal cluster including the left frontal pole, inferior frontal gyrus and orbito-frontal cortex. Performance on concrete verb comprehension, on the other hand, was additionally correlated with lesions in the left angular gyrus, temporal occipital fusiform cortex, occipital fusiform gyrus, and superior and inferior lateral occipital cortex. This cluster also encompassed subcortical structures involving the precuneus cortex, posterior and anterior cingulate gyrus and paracingulate gyrus, and white matter tracts corresponding to inferior longitudinal fasciculus, anterior and posterior segment of the arcuate fasciculus. Similar results were obtained when age, education and time post onset were added to these analyses as covariates. These clusters, however, dropped out of significance when lesion volume was added to this analysis as a covariate.

A direct contrast between the production and comprehension of concrete verb did not reveal any significant clusters except at a lower threshold of *p* < 0.01 voxel-level, FWE cluster-level corrected at *p* < 0.05 (Fig. 5B). The results indicated that the left temporal pole, orbito-frontal cortex, and planum polare were associated with verb naming over-and-above comprehension, whereas regions within the left temporal lobe extending posteriorly into the occipital cortex including posterior middle temporal gyrus, anterior and posterior inferior temporal gyrus, anterior parahippocampal gyrus, temporal occipital fusiform cortex, superior lateral occipital cortex, inferior lateral occipital cortex, posterior cingulate gyrus and precuneus cortex and white matter tracts corresponding to fornix and cingulum were associated with verb comprehension over-and-above naming. When age, education and time post onset were added as covariates to these analyses, similar results were obtained for naming over comprehension with an additional cluster (1597 voxels) covering white matter tracts corresponding to the left anterior segment of the arcuate fasciculus. On the other hand, the cluster associated with comprehension over naming was smaller in size (1594 voxels) covering the same regions excluding the left lateral occipital cortex, cingulate gyrus and precuneus cortex and white matter tracts corresponding the cingulum. These clusters, however, dropped out of significance when lesion volume was added to this analysis as a covariate. Significant clusters and main peak MNI coordinates are shown in Table 6.

**Table 6:** Details of the significant clusters and peak MNI coordinates associated with single-word verb processing based on the naming and picture-to-word matching tests. Anatomical labels obtained using the Harvard-Oxford and natbrainlabs atlases in MNI space.

**Test**				**MNI co-ordinates**		
	**Location**	**Cluster size** (voxels)	**Z**	*x*	*y*	*Z*
Verb naming	Posterior segment of arcuate fasciculus	9220	4.85	− 34	− 52	22
	Cortico-spinal tract		4.79	− 30	− 42	26
	Anterior middle temporal gyrus		4.65	− 56	0	− 22
	Internal capsule		4.58	− 10	− 2	− 6
	Temporal pole		4.50	− 52	10	− 14
	Planum polare		4.37	− 50	− 8	− 22
	Posterior temporal fusiform cortex		4.35	− 44	− 24	− 16
	Posterior inferior temporal gyrus		4.28	− 44	− 42	− 14
	Inferior longitudinal fasciculus		4.08	− 38	0	− 34
	Anterior inferior temporal gyrus		4.04	− 46	− 8	− 34
	Posterior supramarginal gyrus		4.01	− 58	− 42	40
	Posterior middle temporal gyrus		3.94	− 50	− 12	− 18
	Anterior segment of arcuate fasciculus		3.67	− 38	− 14	28
	Orbito-frontal cortex	270	4.45	− 28	20	− 24
	Frontal pole		3.85	− 40	36	2
	Inferior frontal gyrus		3.52	− 46	30	14
Verb comprehension	Posterior inferior temporal gyrus	13,818	5.52	− 46	− 44	− 16
	Planum polare		5.27	− 38	− 16	− 12
	Superior lateral occipital cortex		4.92	− 34	− 66	32
	Cortico-spinal tract		4.84	− 10	− 4	− 6
	Inferior longitudinal fasciculus		4.83	− 46	− 24	− 18
	Anterior inferior temporal gyrus		4.74	− 44	− 8	− 32
	Internal capsule		4.66	− 14	− 20	− 2
	Angular gyrus		4.56	− 32	− 56	22
	Posterior middle temporal gyrus		4.48	− 60	− 14	− 22
	Posterior temporal fusiform cortex	406	4.06	− 36	− 6	− 32
	Posterior segment of arcuate fasciculus		3.80	− 36	− 46	20
	Anterior middle temporal gyrus		3.23	− 60	6	− 16
	Anterior segment of arcuate fasciculus		3.27	− 36	− 32	24
Verb naming > comprehension^a^	Temporal pole	1325	3.57	− 54	12	− 8
	Orbito-frontal cortex		3.50	− 24	20	− 24
Verb comprehension > naming^a^	Inferior temporal gyrus	3643	3.75	− 50	− 58	− 8
	Temporal occipital fusiform cortex		3.81	− 36	− 50	− 12
	Inferior temporal gyrus		3.08	− 42	− 10	− 32
	Anterior middle temporal gyrus		3.02	− 52	− 12	− 24

Results threshold *p* < 0.0005 voxel-level, FWE cluster-level corrected *p* < 0.05.

a Direct contrast: threshold dropped to *p* < 0.01 voxel-level, FWE cluster-level corrected *p* < 0.05.

## 4 Discussion

This is the first large-scale study that examined both the neuropsychological status and neural correlates of processing concrete and abstract verbs in a large group of patients, covering the full range and classifications of post-stroke aphasia. A review of the current aphasiological and neuropsychological assessment batteries identified that there was need for tests to assess single-word verb comprehension. A novel verb semantic comprehension battery was developed, which is sensitive and allows for systematic comparisons across different verb types. This battery includes: (a) a written word synonym judgment test, which was used to examine the comprehension of concrete and abstract verbs, as well as the impact of frequency and the interaction effect between concreteness and frequency; and (b) a picture-to-word matching test that was used in conjunction with the OANB (Druks and Masterson, 2000) to examine the relationship between verb comprehension and production. Importantly, this new battery has a number of key advantages over the existing tests: (i) all items in both tests, including probes, targets and distractors, are verbs; (ii) they are specifically designed to examine verb comprehension deficits; (iii) the synonym judgment test includes both concrete and abstract items; (iv) the synonym judgment test also varies two dimensions (imageability and frequency); and (iv) the picture-to-word matching test included semantically-related distractors, which increases the sensitivity of the tests in detecting semantic deficits. A full list of items for both tests is provided in the Supplementary materials.

The findings revealed an effect of concreteness on verb processing, with the expected advantage of concrete verbs over abstract verbs in post-stroke aphasia. The pattern of individual performance was similar across all patients in this study, with all showing the concreteness effect. These findings are consistent with other neuropsychological studies on healthy adults (e.g., Wiemer-Hastings and Xu, 2005), post-stroke aphasia (e.g., Hoffman et al., 2011b, Sandberg and Kiran, 2014), and semantic dementia (Jefferies et al., 2009), which have shown concreteness effects in noun processing. This study revealed a similar pattern for verbs, which vary in concreteness to a greater degree than nouns and typically have lower values overall. This evidence suggests that a robust concreteness effect is present in aphasia irrespective of word class. It also supports the findings showing that imageability strongly predicts performance and verb deficits in patients with post-stroke aphasia (Bird et al., 2001b, Bird et al., 2003), and another study that showed an imageability effect on verb processing in aphasia using a small number of items (14 verbs) (Dube et al., 2014). The concreteness effect in lexical processing has been explained in the literature with reference to concrete words encompassing richer semantic representations (Jones, 1985), as they are supported by sensory as well as verbal experience (Paivio et al., 1968). In addition, abstract words are contextually more variable (Schwanenflugel and Shoben, 1983). Recent work has quantified contextual variability of words in a measure called semantic diversity (Hoffman et al., 2013), which confirms that abstract words have the tendency to appear in a wider range of contexts. As a result they have more variable meanings and are more demanding for the executive-semantic and language systems (Hoffman, 2015, Hoffman et al., 2015).

The neural correlates associated with verb processing (concrete and abstract verb comprehension, and concrete verb production) were identified using voxel lesion-symptom mapping. The results showed that widespread cortical regions within the left hemisphere were associated with verb semantic comprehension during the synonym judgment task. The areas included left temporal, parietal and frontal regions and lateral occipital cortex, including white matter tracts corresponding to the left inferior longitudinal fasciculus and posterior segment of the arcuate fasciculus. Further analysis revealed that these regions were mainly modulated by word concreteness: left frontal areas were more involved with processing abstract verbs, which mainly refer to thought and emotional verbs (e.g., ‘to cogitate’ and ‘to empathise’). On the other hand, concrete verbs, which mostly refer to action and motion verbs (e.g., ‘to wash’ and ‘to chase’) were supported more by left dorsal posterior temporal and occipital regions and white matter tracts corresponding to posterior inferior longitudinal fasciculus and posterior segment of the arcuate fasciculus. The greater involvement of frontal regions with abstract words suggests that abstract verb processing relies more on language and executive systems, and this has been interpreted in the literature in terms of the role of frontal regions in semantic control, perhaps as a result of the more variable meanings associated with abstract concepts. This view is supported by fMRI (Badre et al., 2005, Hoffman et al., 2015, Noppeney and Price, 2004) and TMS studies (Hoffman et al., 2010, Whitney et al., 2012), as well as studies on patients with inferior frontal gyrus lesions (Bedny et al., 2007, Hoffman et al., 2010). The verbs in our synonym judgment test where presented in their infinitive form preceded with the particle ‘to’ (e.g. ‘to cogitate’); which limits their concept to verbs but it does not fully elucidate the meaning of the abstract concepts, and thus the patients might have to create an appropriate meaningful context that defines the specific semantics of the probe verb and the three choices in order to give a correct response. In contrast, left posterior temporal and ventral temporo-occipital regions were more related to concrete words, suggesting that concrete verbs rely more on the sensory aspect of the semantic knowledge. This could reflect the contribution of sensory experience to concrete concepts, a result which has also been found in previous fMRI and neuropsychological studies (Binder et al., 2005, Hoffman et al., 2015). The results from this study also revealed common areas that support processing both concrete and abstract verbs in the left anterior temporal lobe. The lack of concreteness effect in the anterior temporal lobe has been previously shown in TMS experiments (Pobric et al., 2007) and in semantic dementia (Jefferies et al., 2009). Taken together, the results from this study and previous work provides strong evidence proposing graded differences within the cortex associated with processing concrete and abstract concepts, despite using different word class (nouns versus verbs), experimental group (healthy adults or semantic dementia versus post-stroke aphasia) or methodology (fMRI or TMS versus lesion-symptom mapping).

The current study failed to find any evidence for a frequency effect for verb processing accuracy in post-stroke aphasia. The impact of imageability but not frequency on lexical comprehension has also been shown in studies of patients with semantically-impaired aphasia using nouns (e.g., Franklin, 1989, Hoffman et al., 2011b, Jefferies and Lambon Ralph, 2006), and patients with aphasia using verbs (Bastiaanse et al., 2016). The lack of frequency effect also aligns with some previous studies on brain-injury patients (Kemmerer and Tranel, 2000), while going against findings that have shown an effect of frequency for processing nouns in healthy adults (Balota and Chumbley, 1984) and aphasia (e.g., Cuetos et al., 2002, Nickels and Howard, 1995). Whilst this discrepancy might reflect the word class used, other studies have also found an absent or reserved frequency effect on noun processing in semantically-impaired aphasic patients (e.g., Hoffman et al., 2011a, Hoffman et al., 2011b) and on verb processing in agrammatic and non-fluent aphasia (Berndt et al., 1997, Breedin, 1996, Breedin et al., 1998). There are some plausible explanations to account for the lack of frequency effect in aphasia. Breedin et al. (1998) suggested that low-frequency verbs are easier to retrieve because they are semantically more unique compared to high-frequency verbs that are semantically more complex. Hoffman et al. (2011b) argued that frequency effects were masked by semantic diversity (high-frequency words tend to occur in more diverse contexts and thus are more demanding to process). Berndt et al. (1997) noted that high-frequency words are sometimes omitted in agrammatism, whereas low-frequency words tend to be spared. Our lesion-symptom mapping for the high and low-frequency verbs revealed overlapping regions across the left temporal, parietal, frontal and occipital cortices. These regions are part of the neural correlates associated with the overall performance on the verb synonym judgment test. When directly contrasting high and low frequency verbs, we only obtained significant differences in one direction (high > low frequency). This one-way difference suggests that there may be additional processing required by the high frequency items. The regions identified include anterior temporal, insular frontal regions – which might reflect the additional language and executive demands that previous authors have noted for high over low frequency words (Breedin et al., 1998, Hoffman et al., 2011b).

The production and comprehension of single-word verbs were also examined using the same items, and findings provide evidence of a reliable performance difference with the comprehension advantage during picture-to-word matching task compared to production during picture naming. This is in line with other studies in aphasia (e.g., Berndt et al., 1997, Breedin et al., 1998), and in brain-injured patients with verb deficits (Kemmerer et al., 2012, Kemmerer et al., 2001). These results are expected given the nature of the two tasks; while both tasks require visual recognition of the pictures followed by activation of the corresponding verb concept and mapping to the semantics of the verb, but single-word comprehension and especially matching tasks are constrained by forced-choices, which is less demanding relative to production tasks, including naming (Gainotti et al., 1995). A wide range of common cortical regions were identified in support of both comprehension and production processes of concrete verbs along the left temporal pole, anterior and posterior middle and inferior temporal gyri, posterior temporal fusiform cortex, planum polare, and internal capsule. A direct contrast revealed that the left anterior ventral prefrontal cortex, orbito-frontal cortex and temporal pole were more involved with verb production over comprehension. The involvement of prefrontal cortex with verb production aligns with other neuropsychological studies, which found lesion in ventral prefrontal gyrus associated with impaired performance on action verb tasks (Kemmerer et al., 2012) and verb naming (Tranel et al., 2001), as well as TMS experiments showing an involvement of left prefrontal cortex for action verb naming (Gerfo et al., 2008). In contrast, regions that were more associated with verb comprehension over production included left temporal and occipital regions and white matter tracts corresponding to inferior occipito-frontal fasciculus. These grey and white matter regions correspond to the ventral language pathway and could be involved in the comprehension task because it requires visual recognition of the picture and all the choices, which activates these posterior visual comprehension areas. These regions have been associated with comprehension, recognition and processing of meaning in lesion-symptom mapping (Bates et al., 2003) and fMRI studies (e.g., Saur et al., 2008). Concrete verb comprehension on both picture-to-word matching and synonym judgment tests was associated with posterior lesions in the ventral temporal-occipital regions, and this involvement has been interpreted as an engagement of visual-motion features associated with action verb concepts (e.g., Bendy et al., 2008, Grossman et al., 2002, Kemmerer et al., 2008). This could suggest that successful performance on concrete verb comprehension relies on the ability to comprehend the action patterns associated with a particular verb that is inferred by the static pictorial or written stimulus.

To conclude, a wide range of single-word verb semantic comprehension tests were developed in this study, and these neuropsychological tests were used to provide empirical evidence to reveal performance differences in the comprehension of concrete and abstract verbs, as well as between the comprehension and production of concrete verbs in post-stroke aphasia. This novel verb semantic comprehension battery (provided in the Supplementary materials) is sensitive in detecting semantic deficits, given the significant lower performance of the groups of participants with aphasia in comparisons to the control group on all tests. Although there was no correlation between the performances of the control group and their age on all tests, it should be noted that this control group was not age-matched to the patient group. Therefore, the battery can benefit from future standardisation to be used with different age groups and patient populations. The battery can be used in neuropsychology and psycholinguistic research, and can be utilised in clinical practice during the assessment and diagnostic phases to detect semantic deficits. This could lead to a better planning of intervention programmes across different populations with language impairments, including aphasia, traumatic brain injury and neurodegenerative diseases.

Furthermore, lesion-symptom mapping demonstrated that the neural correlates of single-word verb processing are distributed within wide left cortical regions; with graded differences observed in modalities (production and comprehension), and concreteness (concrete and abstract). These findings align with neuropsychological and neuroimaging studies that have shown similar effects using nouns among healthy adults and patient groups, such as aphasia and semantic dementia. These patterns of performance indicate greater difficulties with the comprehension of abstract verbs compared to concrete verbs, and more impairment with the production of concrete verbs compared to the comprehension of concrete verbs, one might expect that the production of abstract verbs will be similarly challenging. Further research will be able to investigate the production of abstract verbs. This will be harder to examine, given that abstract items cannot be easily represented in pictorial material, and thus the experiment will have to rely on different tasks (such as content analyses of elicited connected speech rather than naming).

## Appendix A A novel verb semantic battery

Supplementary tables Image 1
