# Supplementary material for: The behavioural patterns and neural correlates of concrete and abstract verb processing in aphasia: A novel verb semantic battery
Source: Neuroimage Clin. 2017 Dec 6;17:811–25. doi: 10.1016/j.nicl.2017.12.009 (PMC5883238; doi:10.1016/j.nicl.2017.12.009)
Supplement: Supplementary file 1 — Supplementary tables [file mmc1.pdf]

Supplementary Appendix

First test: Verb synonym judgment

Category	Probe	Target	Distractor 1	Distractor 2
Concrete & High Frequency	**To boil**	To heat	To wound	To comb
	**To fall**	To drop	To twist	To dance
	**To park**	To place	To sweep	To breathe
	**To murder**	To kill	To swallow	To crack
	**To suck**	To sup	To break	To pay
	**To carve**	To shape	To spill	To hunt
	**To spend**	To pay	To exercise	To chase
	**To add**	To count	To rise	To sink
	**To break**	To crack	To plant	To freeze
	**To rise**	To stand	To park	To smile
	**To spill**	To drop	To murder	To telephone
	**To twist**	To spin	To add	To wash
	**To sweep**	To clean	To fish	To heat
	**To brush**	To comb	To fall	To travel
	**To chase**	To hunt	To fry	To brush
	**To sink**	To submerge	To embrace	To place
	**To freeze**	To chill	To carve	To attack
	**To smile**	To grin	To boil	To paint
	**To wash**	To clean	To suck	To enjoy
	**To telephone**	To ring	To spend	To wipe
Concrete & Low Frequency	**To bandage**	To plaster	To vacuum	To ski
	**To shoplift**	To pilfer	To snow	To soothe
	**To hike**	To trek	To snooze	To cuddle
	**To skate**	To ski	To shoplift	To hover
	**To ice**	To frost	To shower	To glue
	**To drum**	To strum	To skate	To sprint
	**To butter**	To lubricate	To mop	To box
	**To fence**	To joust	To iron	To sunbathe
	**To glue**	To paste	To fence	To holiday
	**To snooze**	To nap	To mash	To microwave
	**To mash**	To squash	To ice	To snowball
	**To crochet**	To stitch	To hike	To toast
	**To babysit**	To soothe	To gargle	To toboggan
	**To wallpaper**	To plaster	To garden	To punch
	**To sprint**	To dash	To drum	To uncork
	**To box**	To punch	To defrost	To wallpaper
	**To sunbathe**	To tan	To crochet	To weightlift
	**To microwave**	To irradiate	To bandage	To jog
	**To snowball**	To proliferate	To butter	To saw
	**To toast**	To scorch	To babysit	To parachute

**Continued**				
**Category**	**Probe**	**Target**	**Distractor 1**	**Distractor 2**
Abstract & High frequency	**To permit**	To enable	To cost	To advance
	**To resolve**	To settle	To stay	To suppose
	**To evolve**	To advance	To suggest	To assume
	**To empathise**	To understand	To permit	To benefit
	**To ban**	To prohibit	To quote	To reward
	**To expect**	To suppose	To relieve	To cope
	**To suggest**	To offer	To prefer	To blame
	**To suppose**	To assume	To resolve	To happen
	**To involve**	To include	To mean	To cause
	**To better**	To improve	To keep	To declare
	**To quote**	To cite	To involve	To plan
	**To define**	To describe	To expect	To regard
	**To relieve**	To calm	To evolve	To state
	**To diminish**	To decrease	To empathise	To ease
	**To benefit**	To reward	To diminish	To realise
	**To cost**	To value	To define	To wonder
	**To cope**	To handle	To chose	To decrease
	**To cause**	To generate	To believe	To ban
	**To blame**	To accuse	To need	To better
	**To state**	To declare	To accord	To value
Abstract & Low Frequency	**To implore**	To beseech	To veto	To airbrush
	**To deprive**	To divest	To sadden	To toughen
	**To revere**	To worship	To subdue	To rue
	**To foretell**	To prophesize	To revere	To remit
	**To muse**	To cogitate	To disallow	To redeem
	**To defraud**	To swindle	To rig	To proffer
	**To subdue**	To restrain	To muse	To renege
	**To deviate**	To digress	To cogitate	To under-develop
	**To veto**	To overrule	To impel	To remake
	**To impel**	To coerce	To hone	To fault
	**To disallow**	To prohibit	To foretell	To verbalise
	**To rig**	To equip	To implore	To absolve
	**To partition**	To subdivide	To revert	To peruse
	**To revert**	To regress	To debase	To prophesize
	**To peruse**	To scrutinize	To collate	To deprive
	**To sadden**	To depress	To lease	To defraud
	**To prophesize**	To forecast	To deselect	To extol
	**To hone**	To polish	To deviate	To infer
	**To deselect**	To discard	To cogitate	To covet
	**To cogitate**	To ponder	To misrepresent	To partition

Second tests: Verb picture-to-word matching

Target	Semantic distractor 1	Semantic distractor 2	Unrelated distractor 1	Unrelated distractor 2
**barking**	roaring	growling	skating	jumping
**begging**	requesting	prying	snowing	ringing
**bending**	crouching	squatting	raking	carrying
**biting**	eating	chewing	knocking	watching
**bleeding**	dripping	flowing	washing	skating
**blowing**	puffing	sucking	driving	writing
**bouncing**	juggling	throwing	kissing	skiing
**building**	plastering	creating	diving	tying
**carrying**	taking	lifting	stopping	sinking
**catching**	passing	throwing	kneeling	skipping
**climbing**	falling	jumping	biting	ironing
**combing**	cutting	brushing	riding	stopping
**cooking**	stirring	dining	jumping	dreaming
**crawling**	walking	creeping	planting	melting
**crossing**	running	driving	raining	lighting
**crying**	laughing	sulking	pouring	marching
**cutting**	tearing	ripping	bouncing	weaving
**dancing**	holding	hugging	sleeping	washing
**digging**	hammering	drilling	touching	crying
**diving**	swimming	sinking	running	biting
**drawing**	painting	colouring	pulling	bouncing
**dreaming**	imagining	thinking	combing	fishing
**drilling**	puncturing	digging	walking	shaving
**drinking**	eating	spitting	sewing	raking
**dripping**	pouring	dropping	begging	smoking
**driving**	riding	cycling	tickling	stroking
**dropping**	catching	throwing	shooting	painting
**eating**	drinking	crunching	climbing	drilling
**fishing**	shooting	catching	lighting	singing
**floating**	swimming	sinking	stirring	barking
**flying**	floating	cruising	drilling	knocking
**folding**	wrapping	tearing	licking	roaring
**ironing**	hanging	drying	dropping	climbing
**juggling**	catching	balancing	yawning	sleeping
**jumping**	climbing	skipping	floating	shooting
**kicking**	scoring	throwing	rocking	pulling
**kissing**	licking	loving	flying	sewing
**kneeling**	bending	crouching	shaving	playing
**knitting**	weaving	sewing	crossing	laughing
**knocking**	ringing	tapping	watering	begging

**Continued**				
**Target**	**Semantic distractor 1**	**Semantic distractor 2**	**Unrelated distractor 1**	**Unrelated distractor 2**
**laughing**	crying	smiling	ironing	knitting
**leaning**	bending	standing	writing	stirring
**licking**	eating	drooling	tying	dripping
**lighting**	extinguishing	flashing	building	reading
**marching**	striding	fighting	melting	pointing
**melting**	heating	freezing	kicking	running
**opening**	pulling	knocking	digging	walking
**painting**	drawing	colouring	dancing	rocking
**peeling**	chopping	removing	weighing	skating
**pinching**	gripping	poking	roaring	weighing
**planting**	watering	digging	knitting	sneezing
**playing**	stacking	building	eating	yawning
**pointing**	waving	aiming	folding	kicking
**posting**	giving	receiving	bending	licking
**pouring**	dripping	watering	sliding	blowing
**praying**	requesting	worshiping	weaving	diving
**pulling**	pushing	taking	watching	tickling
**pushing**	sending	pulling	cutting	kissing
**raining**	snowing	hailing	pointing	swinging
**raking**	sweeping	hoovering	drinking	praying
**reading**	writing	scanning	dripping	folding
**riding**	driving	jumping	typing	kneeling
**ringing**	shaking	calling	sailing	flying
**roaring**	barking	howling	carrying	pushing
**rocking**	swinging	shaking	painting	posting
**running**	walking	racing	juggling	cooking
**sailing**	swimming	sinking	crying	crossing
**sewing**	knitting	weaving	reading	touching
**shaving**	cutting	washing	playing	peeling
**shooting**	fishing	hitting	barking	combing
**singing**	talking	dancing	crawling	cutting
**sinking**	swimming	falling	leaning	bending
**sitting**	crouching	lying	singing	planting
**skating**	skiing	sliding	catching	building
**skiing**	sliding	skating	cooking	waving
**skipping**	jumping	swinging	bleeding	drinking
**sleeping**	dreaming	waking	sinking	raining
**sliding**	slipping	falling	opening	typing
**smiling**	laughing	pouting	fishing	opening
**smoking**	lighting	coughing	marching	sliding
**sneezing**	coughing	blowing	posting	snowing

**Continued**				
**Target**	**Semantic distractor 1**	**Semantic distractor 2**	**Unrelated distractor 1**	**Unrelated distractor 2**
**snowing**	raining	hailing	peeling	riding
**stirring**	dining	spooning	pinching	catching
**stopping**	parking	blocking	dreaming	eating
**stroking**	touching	poking	praying	digging
**swimming**	diving	snorkelling	waving	sitting
**swinging**	flying	skipping	ringing	drawing
**tickling**	touching	itching	pushing	watering
**touching**	pinching	tapping	skipping	juggling
**tying**	opening	stitching	swimming	pouring
**typing**	writing	pressing	skiing	leaning
**walking**	marching	running	stroking	smiling
**washing**	watering	wiping	swinging	crawling
**watching**	listening	observing	drawing	floating
**watering**	sowing	spilling	blowing	dancing
**waving**	pointing	saluting	smiling	driving
**weaving**	knitting	sewing	laughing	swimming
**weighing**	dividing	measuring	smoking	pinching
**writing**	typing	drawing	sneezing	bleeding
**yawning**	sleeping	coughing	sitting	dropping

Please cite the paper when using the tests above. Thank you.
